# Sources of alcohol and associations with drinking frequency and binge drinking among a large sample of adolescents

**DOI:** 10.1016/j.abrep.2026.100690

**Published:** 2026-03-20

**Authors:** Mahmood R. Gohari, Karen A. Patte, Mark A. Ferro, Scott T. Leatherdale

**Affiliations:** a Department of Health Sciences, Brock University, St. Catharines, ON, Canada; b School of Public Health Sciences, University of Waterloo, Waterloo, ON, Canada

**Keywords:** Youth, Substance use, Underage drinking, Alcohol supply, Binge drinking

## Abstract

**Background:**

Underage drinking remains a major public health concern worldwide, contributing to a wide range of health and social harms. While adolescent alcohol use has been widely studied, there is limited evidence on how adolescents obtain alcohol and how these sources relate to drinking behaviours.

**Methods:**

This cross-sectional analysis used data from 8,507 Grades 9–12 students participating in the 2022–2023 COMPASS study across 66 schools in Canada. Analyses were restricted to students who reported alcohol use in the past 12 months. Proportional odds mixed models assessed associations between alcohol sources and two ordinal outcomes: drinking frequency and binge drinking frequency, adjusting for individual- and school-level covariates.

**Results:**

Parents/guardians were the most frequently reported source of alcohol (32%), followed by parties or events (22%) and purchasing or receiving alcohol from others (17%). Parental provision was most common among occasional or infrequent drinkers, whereas commercial sources (stores, restaurants, or bars) were prominent among frequent drinkers and binge drinkers. Adjusted models showed that obtaining alcohol from commercial sources was associated with a more than a fivefold increase in the odds of frequent and binge drinking compared with parental supply.

**Conclusions:**

These findings highlight distinct patterns in how Canadian adolescents report accessing alcohol, underscoring the importance of prevention strategies that address both social and commercial sources. Interventions focused on family environments, party settings, and retail access may help reduce opportunities for alcohol acquisition and mitigate the risk of higher-risk drinking behaviours among youth.

## Highlights

• Parents/guardians were the most common main source of alcohol among adolescents.
• Commercial and peer-related sources were linked to higher-risk drinking.
• Adolescents with the highest binge drinking odds reported obtaining alcohol from stores as their main source.
• Younger adolescents most often obtained alcohol from parents or without permission.
• Findings highlight distinct risk pathways informing targeted prevention strategies.

## 1 Introduction

Underage alcohol consumption remains a major public health concern worldwide, contributing to a wide range of adverse health and social outcomes. Adolescents who drink are at increased risk of developing alcohol dependence later in life (Hingson, Heeren, & Winter, 2006), experiencing academic difficulties (Ellicksonet al., 2003, Patteet al., 2017), engaging in violent behaviours (Ellickson et al., 2003), and facing mental disorders (Kuntscheet al., 2017, Nunes, 2023, Steinfeld and Torregrossa, 2023). Despite widespread legal restrictions on alcohol sales to minors, underage drinking persists, often sustained by various social and commercial sources.

The source from which adolescents obtain alcohol plays a crucial role in shaping their drinking behaviours, including frequency, quantity, and the likelihood of riskier drinking. Social sources, such as friends or peers, have been linked to heavier drinking episodes (Rosenquistet al., 2010, Kuntscheet al., 2017). Parental supply remains complex, with mixed findings: while some evidence suggests that adolescents given alcohol by parents are more likely to drink regularly (McMorris, Catalano, Kim, Toumbourou, & Hemphill, 2011), other studies suggest that parental provision in controlled contexts may mitigate harmful effects (Bellis et al., 2007). Commercial acquisition through liquor stores, bars, or restaurants is often associated with higher overall consumption and more frequent binge drinking (Bellis et al., 2007). These distinct pathways underscore the importance of understanding not just whether adolescents drink, but how they access alcohol.

Identifying and characterizing sources of alcohol among youth is essential for developing effective prevention strategies. Tailored interventions are needed to address different pathways of access, whether through social, parental, commercial, or illicit means. Although adolescent drinking behaviours have been widely studied, little is known about how Canadian youth obtain alcohol or how these sources vary across regions, social contexts, or demographic groups. According to the 2023–24 Canadian Student Alcohol and Drugs Survey (CSADS), among students who reported alcohol use in the past year, the most frequently cited sources were a parent with permission (34%), parties or events (18%), and buying or obtaining alcohol from friends or family (14%) (Health Canada, 2025). Moreover, over two thirds of Canadian students reported that alcohol is fairly or very easy to obtain, with 81% indicating access through social connections such as friends or family (Health Canada, 2025). However, few studies have examined how these sources relate to adolescents’ actual drinking patterns and behaviours.

Building on this gap, the present study examines the sources of alcohol among adolescents and assesses how these sources are associated with drinking and binge drinking behaviours. Specifically, we examine: (1) To what extent do adolescents obtain alcohol from different sources, and how do these sources vary across contexts or subgroups? And (2) How are adolescents’ drinking behaviours related to the different sources from which they obtain alcohol? Additionally, we investigate whether the distribution of reported alcohol sources varies by contextual factors, such as province, urbanicity, and number of alcohol outlets, and individual characteristics, such as age, gender, and family affluence.

## 2 Methods

This cross-sectional study used survey data collected during the 2022–23 school year from students in grades 9–12 who participated in the COMPASS study, a large-scale, ongoing longitudinal research project focused on youth health behaviours. The full COMPASS sample for that year included students from participating secondary schools across three provinces. For the present analysis, we restricted the sample to students who reported consuming alcohol at least once in the past 12 months, resulting in an analytic subsample of 8,507 students. Students were recruited from 66 secondary schools across three Canadian provinces, British Columbia, Ontario, and Prince Edward Island, as part of the COMPASS study. The minimum legal age for purchasing alcohol in all three provinces is 19 years. COMPASS employed an active-information, passive-consent parental permission protocol, in which parents were informed about the study in advance and given the opportunity to withdraw their child; if no withdrawal was made, student participation was based on student assent to participate where they were provided the opportunity to decline participation at any point in time; an approach shown to support higher response rates and reduce sampling bias in school-based substance use research (Chartier et al., 2008). The COMPASS study received ethics clearance from the University of Waterloo Office of Research Ethics Board.

### 2.1 Measures

**Source of Alcohol**: Participants were asked: “In the last 12 months, how did you usually get the alcohol you consumed? (Mark only one).” The original survey question included the following response options: (1) I took it from someone else without permission; (2) A parent or guardian gave it to me; (3) I got or bought it from a friend or family member (not a parent or guardian); (4) I got or bought it from someone else; (5) It was shared at a party; (6) I got or bought it at a public event (e.g., concert or sporting event); (7) I bought it or someone bought it for me at a liquor store; (8) I bought it or someone bought it for me at a convenience store; (9) I bought it or someone bought it for me at a grocery store; (10) I bought it or someone bought it for me at a restaurant or bar; and (11) Other. For analysis, these responses were grouped into six categories: (1) Took without permission: took alcohol from someone, including family or friends, without permission; (2) Parent/guardian: a parent or guardian provided the alcohol; (3) Bought/receiving from others: obtained or purchased alcohol from a friend, family member (not a parent/guardian), or someone else; (4) Party/event: alcohol was shared or obtained at a party or public event; (5) Commercial sources: the adolescent purchased alcohol, or someone purchased it for them, at a liquor, convenience, or grocery store, or at a restaurant or bar; and (6) Other.

**Alcohol consumption**: Alcohol use was assessed using two self-reported items on past-year drinking and binge drinking. Drinking frequency was measured with the question: “In the last 12 months, how often did you have a drink of alcohol that was more than just a sip?” Responses were provided on a 10-point scale ranging from “I have never drunk alcohol” to “every day.” Because this study included only adolescents who reported alcohol consumption, the “never use” category was not present. Due to the low frequency of responses in the upper categories, responses were collapsed into three groups: occasionally (less than once a month), infrequently (1–3 times per month), frequently (one or more time per week).

In the COMPASS survey, binge drinking was defined as consuming five or more standard drinks on one occasion, consistent with national Canadian youth surveillance indicators that apply a 5+ drinks threshold (Health Canada, 2025). Participants were asked “In the last 12 months, how often did you have 5 drinks of alcohol or more on one occasion?” Responses on the original 8- category scale were recoded as no binge drinking (none), occasionally (less than once a month), infrequently (1–3 times per month), and frequently (one or more than one time per week) binge drinking.

**Student-level covariates:** Participants reported their gender, age, ethnicity, and relative family economic status. Gender (categorized as cisgender boy, cisgender girl, or gender diverse) was derived from participants’ self-reported sex assigned at birth (male, female; responses of “prefer not to say” were coded as missing) and their reported gender identity (girl/woman, boy/man, non-binary, Two-Spirit, described differently, or prefer not to say). Age was categorized into three groups: 13–14 years, 15–16 years, and 17–18 years old. Ethnicity was categorized as Asian, Black, White, or Another/Multi-ethnic. Socioeconomic status was measured using a subjective rating of relative economic position by asking students whether they perceived their family to be more, less, or as comfortable financially compared to the families of other students in their class.

**School-level characteristics:** School-level characteristics were collected in relation to urbanicity (rural/small urban, medium urban, large urban) and the number of alcohol outlets around schools. The data on urbanicity were obtained from the 2021 Canadian census data (Statistics Canada, 2021). Urban and rural categories were based on Statistics Canada’s Statistical Area Classification system and were derived from school postal codes. Large urban areas were defined as census divisions with populations over 100,000. Medium urban areas had populations between 30,000 and 99,999, and small urban areas had populations under 30,000. Due to the small sample size from medium urban areas, urbanicity was reclassified into three categories: rural or small urban, medium urban, large urban areas.

We quantified alcohol outlet density as the number of off-premises outlets within a 10 km buffer around schools, following the approach used in previous studies (Gohari, Cook, Dubin, & Leatherdale, 2021). A 10 km buffer was selected to represent a realistic travel distance within which adolescents may access alcohol retailers. This distance can generally be travelled within 10–15 min by car and reflects a geographic range within which older adolescents may drive themselves, obtain rides from peers, or otherwise access commercial outlets, particularly outside of school hours. Locations of off-premise alcohol outlets were obtained from Enhanced Points of Interest (EPOI) databases (DMTI Spatial Inc, 2017); these vector GIS databases provide information on the location of specific services and businesses for all provinces/territories of Canada.

### 2.2 Statistical methods

To address the research objectives, we explored the distribution and proportion of each alcohol source within the sample. These results are further presented according to individual-level characteristics (age, gender, economic position, and ethnicity) and school-level characteristics (province and urbanicity). To examine the association between sources of alcohol and adolescent drinking behaviours, we fit proportional odds models using the cumulative link mixed model framework. Two separate models were estimated, one with drinking level as the outcome and the other with binge drinking level as the outcome. Both outcomes were measured as ordered categories reflecting increasing frequency of use. The models included individual- and school-level characteristics. Models accounted for the hierarchical structure of the data by including school-level random intercepts to capture clustering of students within schools. Results are reported as adjusted odds ratios (aOR) with 95% confidence intervals, representing the relative odds of reporting a higher category of drinking behaviour associated with each alcohol source and covariate, after adjusting for other factors in the model. All analyses were conducted using SAS Studio (Enterprise Edition, version 3.82), and statistical significance was evaluated at a p-value of <0.05.

**Missing Values.** Of the total 8,507 participants, 92.5% (n = 7,936) had complete data, 3.7% (n = 314) had one missing value, 3.0% (n = 259) had two missing values, and 0.5% (n = 44) had more than two missing values. The extent of missing data varied across variables. Missing rates ranged from 0.2% for age to 2.7% for reports of ethnicity. Because of low missing rates we did not use any imputation methods and used pairwise deletion approach.

## 3 Results

**Fig. 1:**
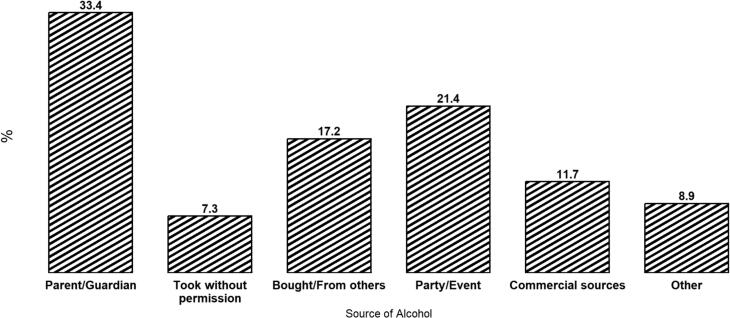
Primary source of alcohol to Canadian adolescents participating in COMPASS study, 2022–23 school year. Percentages indicate the proportion of respondents identifying each source as their primary source of obtaining alcohol.

The study sample included adolescents, with 53.1% identifying as cisgender girl and 69.8% as White. The mean age of participants was 15.89 years (SD = 1.26) (Table 1). Fig. 1 indicates that the most commonly reported primary source of alcohol was a parent/guardian, reported by over 30% of respondents in each group. The second most commonly reported source was parties and events.

**Table 1:** Demographic and behavioural characteristics of Canadian adolescents participating in the COMPASS study, 2022–23 school year.

Characteristics	%	N
Gender		
Cisgender girl	53.1	4504
Cisgender boy	41.6	3532
Transgender and gender diverse	5.3	445
Age		
13–14 year old	14.6	1244
15–16 years old	50.1	4289
17–18 years old	35.3	3021
Ethnicity		
Asian	2.9	240
Black	3.1	257
Latin American	3.8	315
White	69.8	5829
Multiethnic	9.9	825
Another category	10.5	880
Perceived relative affluence		
More comfortable	33.6	2876
As comfortable	54.4	4647
Less comfortable	12.0	1027
Alcohol retailers in 10 km buffer		
1	10.9	931
2–5	43.9	3762
6 or more	45.2	3882
Urbanicity		
Rural/small urban	30.8	2644
Medium urban	26.1	2241
Large urban	43.0	3690
Province		
British Columbia	8.9	766
Ontario	78.4	6726
Prince Edward Island	12.7	1083
Drinking level		
Occasionally (less than once a month)	38.0	3257
Infrequently (1–3 times a month)	43.6	3741
Frequently (1+ times a week)	18.4	1577
Binge drinking level		
None	35.0	2974
Occasionally (less than once a month)	29.3	2489
Infrequently (1–3 times a month)	26.3	2240
Frequently (1 + a week)	9.4	804

**Fig. 2:**
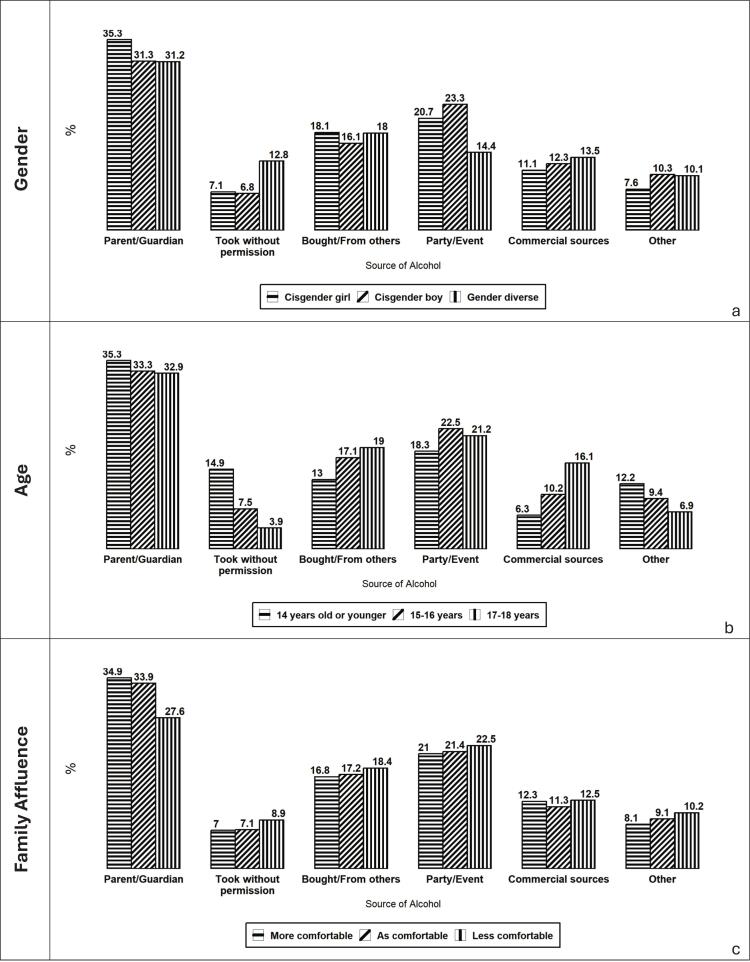
Primary source of alcohol to Canadian adolescents participating in COMPASS study by gender identity (a), age group (b), and perceived family affluence (c), 2022–23 school year. Values represent the percentage of adolescents reporting each source.

Fig. 2 illustrates variation in reported alcohol sources across gender, age, and perceived family affluence. Cisgender girls are more likely than cisgender boys and gender-diverse adolescents to report parents/guardians as their primary source of alcohol, whereas gender-diverse youth are less likely to obtain alcohol at parties or events. Clear age differences are observed: younger adolescents are substantially more likely to report taking alcohol without permission and rely more heavily on parents/guardians, while commercial sources become more prominent among older adolescents. Differences by perceived family affluence are less pronounced; however, adolescents reporting lower affluence are somewhat less likely to identify parents/guardians as their primary source compared to their more affluent peers.

**Fig. 3:**
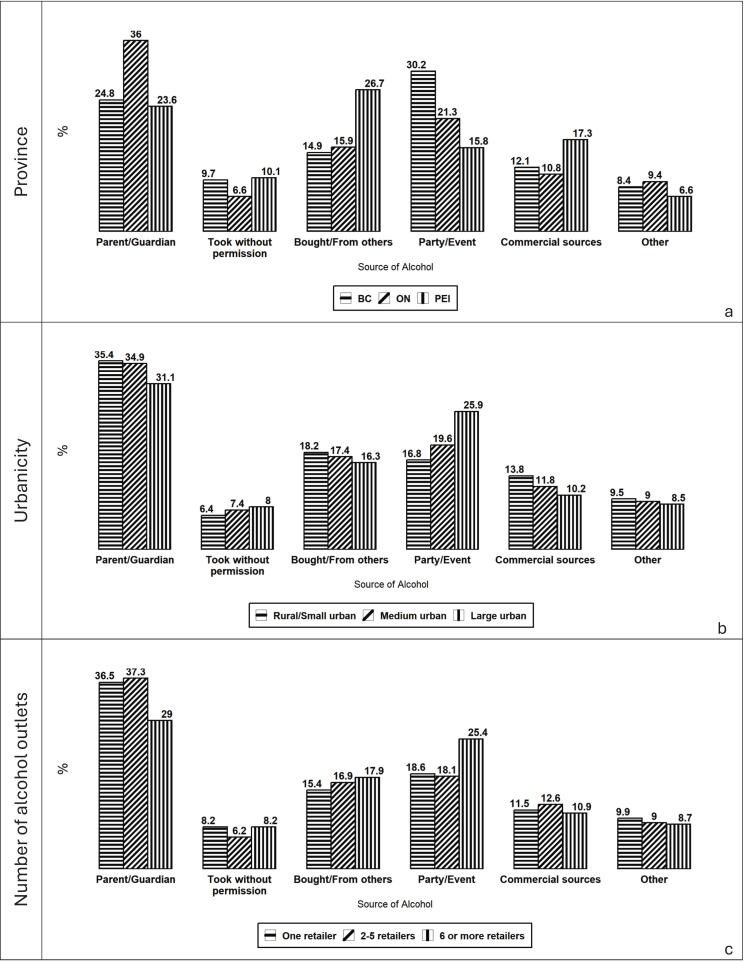
Primary source of alcohol to Canadian adolescents participating in COMPASS study by province (a), rurality/urbanicity (b), and alcohol retailer density (c, 2022–23 school year. Values represent the percentage of adolescents reporting each source.

Fig. 3a indicates that alcohol sources vary by province. In Ontario, parents/guardians are the most common source (36.0%), while in British Columbia, parties or events lead (30.2%), followed by parents/guardians (24.8%). In Prince Edward Island, buying or receiving alcohol from others is most frequent (26.7%), roughly double the proportion in Ontario and British Columbia.

Fig. 3b shows that parents/guardians are the primary source across all rural and urban settings, with slightly higher proportions in rural or small-urban areas. Parties or events are the second most common source, particularly in large urban areas (25.8%) compared with medium (18.9%) and rural or small communities (16.3%).

Fig. 3c demonstrates that adolescents in areas with six or more alcohol retailers are less likely to report parents/guardians as their source (29.0%) than those in areas with fewer retailers (36.5–37.3%). Conversely, youth in high-retailer areas more often obtain alcohol at parties or events.

**Fig. 4:**
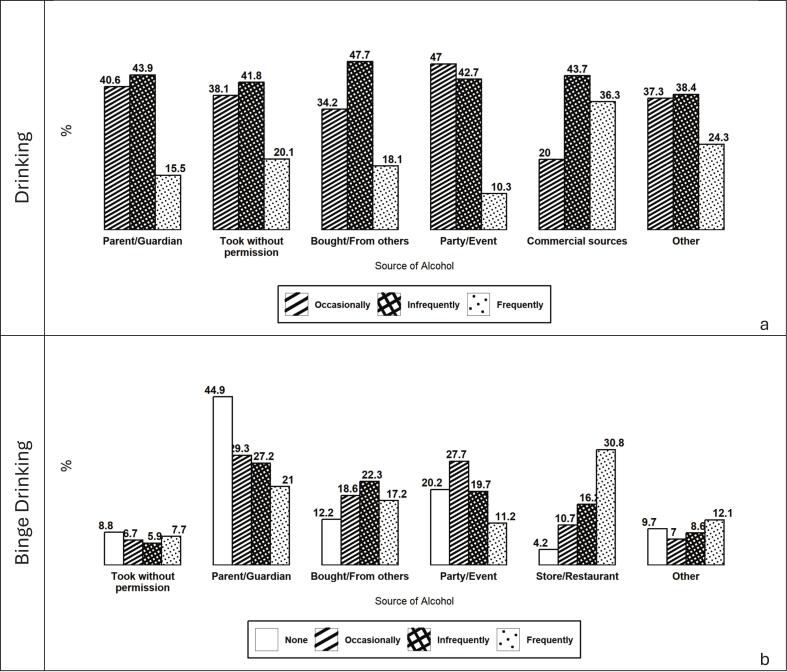
Association between primary source of alcohol and drinking and binge drinking behaviours of Canadian adolescents participating in COMPASS study (2022–23 school year) by drinking frequency (a) and binge drinking frequency (b). Values represent the percentage of adolescents reporting each source.

Fig. 4a illustrates differences in alcohol sources by drinking frequency. Parents/guardians are the most commonly reported source across all drinking levels, although their relative contribution declines as drinking becomes more frequent (35.7% among occasional drinkers vs. 28.2% among frequent drinkers). Parties or events are most prominent among occasional drinkers (26.5%) and decrease with higher drinking frequency. In contrast, commercial sources increase markedly with frequency, accounting for 23.1% among frequent drinkers compared with 6.2% among occasional drinkers.

Fig. 4b shows similar patterns for binge drinking. Adolescents who do not report binge drinking most commonly obtain alcohol from parents/guardians. Among occasional binge drinking, parental provision (29.3%) and parties or events (27.7%) are the leading sources. For infrequent binge drinking, parents remain the primary source (27.2%), followed by obtaining alcohol from others (22.3%). In contrast, commercial sources are most common among frequent binge drinking (30.8%), surpassing parental provision (21.0%).

Table 2 presents the results from the proportional odds model examining the association between sources of alcohol, demographic factors, and levels of binge drinking. Odds ratios greater than 1 indicate higher odds of being in a more frequent binge drinking category compared to the reference group.

**Table 2:** Adjusted odds ratios examining the association between sources of alcohol and drinking and binge drinking behaviours among Canadian adolescents participating in COMPASS study, 2022–23 school year.

Source of alcohol	Drinking	Binge drinking
Parent/guardian: reference group	1.0	1.0
Without permission	1.31 (1.11–1.56)	1.50 (1.26–1.77)
Bought or receiving from others	1.32 (1.17–1.48)	2.34 (2.08–2.64)
Party/Event	0.80 (0.71–0.89)	1.54 (1.38–1.72)
Commercial sources	3.00 (2.61–3.46)	5.18 (4.51–5.95)

Note: Values are adjusted odds ratios (aOR) with 95% confidence intervals, estimated from proportional odds models controlling for individual-level characteristics (age, gender, family affluence, and ethnicity) and school-level characteristics (province and urbanicity), while accounting for the hierarchical structure of students nested within schools. A higher aOR represents greater relative odds of reporting a higher category of drinking.

After adjusting for individual- and school-level characteristics, adolescents who obtained alcohol without permission (aOR = 1.31, 95% CI: 1.11–1.56), bought or received it from others (aOR = 1.32, 95% CI: 1.17–1.48), or accessed it through commercial sources (aOR = 3.00, 95% CI: 2.61–3.46) had significantly higher odds of reporting more frequent drinking compared to those who obtained alcohol from parents/guardians (reference group). In contrast, adolescents who reported accessing alcohol at parties or events had significantly lower odds of frequent drinking compared to the reference group (aOR = 0.80, 95% CI: 0.71–0.89). For binge drinking, the pattern was more consistent: adolescents who obtained alcohol from any source other than parents/guardians showed significantly higher odds of being in a more frequent binge drinking category. Adolescents who identified commercial outlets as their main source were particularly likely to report higher levels of binge drinking.

## 4 Discussion

This study examined the primary sources of alcohol among Canadian adolescents and their associations with drinking frequency and binge drinking. Across the sample, parents/guardians emerged as the most common primary source of alcohol, particularly among adolescents who reported occasional or infrequent drinking. Sources, however, varied across subgroups: commercial sources were more prominent among older youth and those drinking more frequently, while parties and public events were a common source for adolescents who reported occasional drinking and binge drinking.

We found that adolescents’ drinking behaviours varied substantially depending on their source of alcohol, which aligns with prior literature demonstrating that the way adolescents access alcohol is closely tied to their drinking behaviours(Kuntscheet al., 2017, McMorriset al., 2011, Rosenquistet al., 2010). Adolescents who obtained alcohol from peers, parties, or commercial outlets had significantly higher odds of reporting more frequent binge drinking, suggesting that alcohol source is closely associated with higher-risk drinking patterns. Similar trends were observed for overall drinking frequency, although adolescents who primarily obtained alcohol from parties or events reported lower drinking frequency than those who received alcohol from parents/guardians. These findings highlight differences in drinking patterns across alcohol sources and support prevention and policy strategies that address high-risk contexts such as parties and commercial access.

Parents/guardians were the most frequently reported primary source of alcohol among adolescents in this study. Parental provision of alcohol has been linked to several factors, including older parental age, higher parental drinking frequency, and permissive attitudes toward underage drinking or limited awareness of alcohol guidelines for minors (Booth, McCausland, Stafford, Kennington, & Pettigrew, 2023). Although we have no data directly from parents, previous research suggests that many view allowing adolescents to drink at home under supervision as a harm-reduction strategy, believing that doing so provides a safer environment and helps youth learn to drink responsibly (Bowden et al., 2024, Gilligan and Kypri, 2012, Van der Kruket al., 2023). However, evidence consistently shows that parental supply, even in small amounts, does not protect against harm and is associated with greater risk of future problematic drinking (Degenhardtet al., 2015, Sharminet al., 2017). Parental provision may normalize drinking, reduce adolescents’ perceived risks, and indirectly facilitate access to alcohol through broader social networks (Mattick et al., 2018). Further, adolescents who consume alcohol at home with their parents/guardians are more likely to engage in drinking in other settings and to do so more frequently than those who do not drink under parental supervision (Degenhardt et al., 2015). These findings suggest the need for targeted public health messaging to challenge misconceptions around “safe” supervised drinking and to raise awareness about the evidence-based risks and long-term consequences of parental alcohol provision. Given the prominent role of parental supply, prevention strategies should also prioritize parent-focused interventions, including clear guidance on the risks of supplying alcohol to minors, social norm campaigns addressing misconceptions about supervised drinking, and community-level education on hosting alcohol-free gatherings.

Beyond the family environment, social contexts such as parties play a central role in youth alcohol access and consumption. Our findings indicate that parties are the second most common source overall, particularly in larger urban areas and regions with a higher density of alcohol outlets. This observation aligns with prior studies demonstrating that parties are among the most frequent settings where adolescents obtain and consume alcohol, especially in early adolescence (Degenhardtet al., 2015, Paschallet al., 2007). These environments often lack adult supervision, foster peer pressure, and promote rapid or excessive drinking (Bähler et al., 2014, Coxet al., 2019, Poweret al., 2005). In one study of teen-hosted parties, 70% of youth reported that their parents definitely knew alcohol was present, 24% said their parents probably knew, and only 5% said their parents did not know (Friese & Grube, 2014). These findings indicate that parties are not always unsupervised settings; rather, they often occur with parental awareness, underscoring the need for prevention approaches that actively engage parents. Public health efforts should focus on informing parents about their social responsibilities when minors consume alcohol in their homes and increasing understanding of how parents can help keep teen parties alcohol-free.

At a broader structural level, commercial outlets, including liquor stores, convenience stores, bars, and restaurants, also contribute substantially to adolescent alcohol access. Although the legal purchasing age is 19 across all participating provinces, a considerable proportion of 17–18-year-olds reported obtaining alcohol from commercial outlets. This pattern may reflect challenges in enforcing age-of-sale laws and ensuring retailer compliance. For example, Milam, Furr-Holden, Nesoff, and Trangenstein (2021) found that underage buyers were able to successfully purchase alcohol in 68.1% of attempts in U.S. retail stores. Commercial sources were reported more frequently by adolescents who engaged in regular or binge drinking, consistent with research linking retail access with higher levels of alcohol use among youth. These associations reflect not only physical availability but also greater exposure to alcohol marketing and normalization of drinking in retail environments (Martín-Turrero, Valiente, Pastor, Bilal, & Sureda, 2024). This underscores the importance of addressing retail-level accessibility in prevention and policy initiatives, including stricter age-verification practices, targeted compliance checks, and restrictions on marketing and outlet density near schools. Strengthening these measures could help reduce commercial pathways that facilitate underage drinking and associated harms.

While this study focused on adolescents’ primary reported source of alcohol, it is important to recognize that many youth likely access alcohol through multiple channels depending on the situation or availability (Friese, Grube, & Moore, 2013). Adolescents may, for instance, obtain alcohol from parents for family occasions but rely on peers, parties, or commercial outlets in social contexts where parental access is unavailable. Prior research suggests that young people adapt their methods of obtaining alcohol based on opportunity, social networks, and environmental access, often combining social and commercial pathways over time (Chenet al., 2009, Frieseet al., 2013). This overlapping use of sources may be associated with more frequent drinking and binge drinking. Future studies should therefore consider multi-source access patterns to better capture the complexity of underage drinking behaviour and to inform prevention efforts that address both the range and interaction of alcohol sources.

This study benefits from the large, multi-provincial COMPASS dataset and the use of multilevel modeling to account for clustering within schools. However, limitations should be acknowledged. The data are cross-sectional, precluding causal inference about the direction of associations between source of alcohol and drinking levels. Self-reported measures may be subject to recall or social desirability bias, particularly concerning sensitive behaviours such as alcohol use. In addition, while we collapsed sources of alcohol into meaningful categories, this approach may mask nuanced differences within each group (e.g., differences between convenience stores versus restaurants). Further, in this study participants were asked to report only one primary source of alcohol. As adolescents may obtain alcohol through multiple channels depending on context and opportunity, restricting responses to a single source may not fully capture the complexity of alcohol access behaviours. Lastly, the COMPASS study was not designed to be nationally representative, however, the large sample size and diversity of participating students and schools enhance the generalizability of the findings. We therefore expect that the observed patterns would be similar to those found in more representative studies.

## 5 Conclusion

This study shows that most adolescent alcohol access occurs through socially facilitated or permissive channels, including parents, friends, and social gatherings, although adolescents likely rely on multiple sources depending on opportunity and context. Higher levels of drinking are more commonly reported among youth who identify commercial outlets or party environments as their primary source of alcohol. These findings underscore the need for prevention strategies that address both social norms supporting underage drinking and commercial pathways that enable access. Public health efforts should include parental education to challenge assumptions about safe supervised drinking, community-based initiatives to reduce opportunities for alcohol use in party settings and strengthened retail compliance with age-of-sale laws. Addressing multiple access pathways is essential to reducing escalation of risky drinking and associated harms among youth.

## CRediT authorship contribution statement

**Mahmood R. Gohari:** Writing – review & editing, Writing – original draft, Visualization, Methodology, Formal analysis, Data curation, Conceptualization. **Karen A. Patte:** Writing – review & editing, Funding acquisition. **Mark A. Ferro:** Writing – review & editing. **Scott T. Leatherdale:** Writing – review & editing, Funding acquisition.

## Declaration of competing interest

The authors declare that they have no known competing financial interests or personal relationships that could have appeared to influence the work reported in this paper.

## Data Availability

Data will be made available on request.
